# Supplementary material for: Dissociation of salts in water under pressure
Source: Nat Commun. 2020 Jun 16;11:3037. doi: 10.1038/s41467-020-16704-9 (PMC7298052; doi:10.1038/s41467-020-16704-9)
Supplement: Supplementary file 1 — Supplementary Information [file 41467_2020_16704_MOESM1_ESM.pdf]

Supplementary Information for

Dissociation of salts in water under pressure

Zhang et al.

Supplementary Note 1. Convergence of free energy surface at ambient conditions

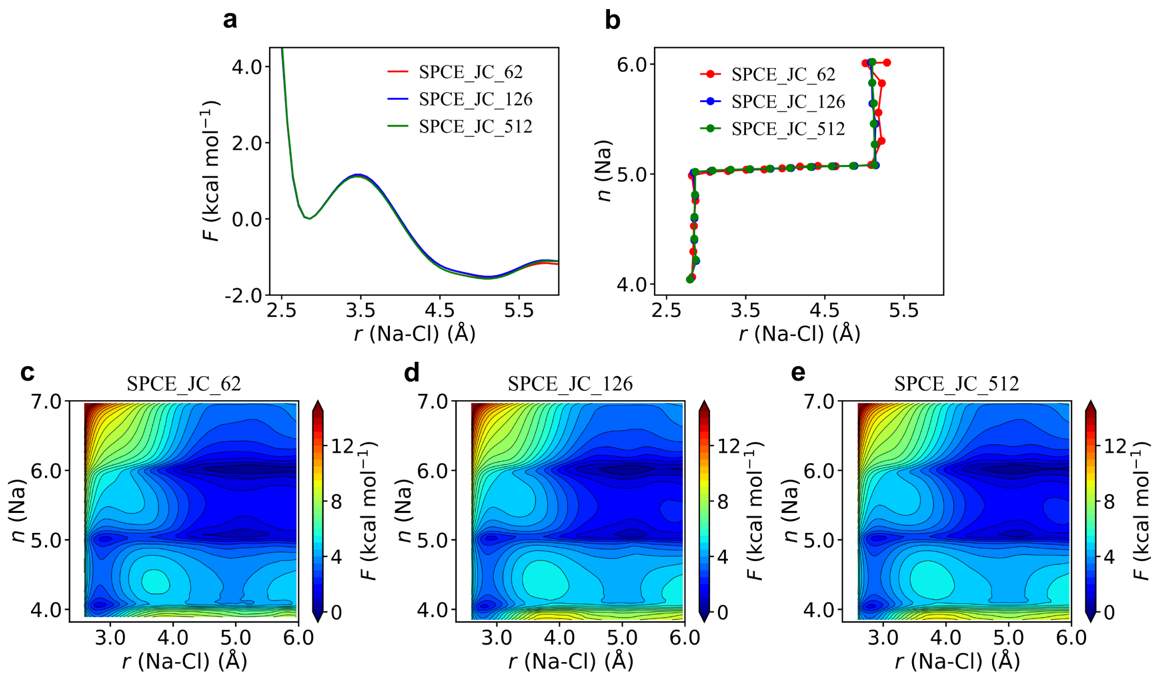

Supplementary Figure 1. Convergence of free energy surface with system size at ambient conditions. One (a, $F(r)$) and two (c-e, $F(r,n)$) dimensional free energy surfaces (FES) of NaCl in water obtained using the SPCE_JC force-field at 300 K and 1 atm, using different simulation cell sizes. (b) Minimum free energy paths obtained for the 2D FESs shown in (c-e). r is the Na-Cl distance and n is the Na-water coordination number.

Using MD simulations with the SPCE_JC force-field, we tested the convergence of the computed free energy surfaces (FES) with respect to salt concentration (cell size) at 300 K and 1 atm. Supplementary Figure 1 shows the FESs calculated for cells with one NaCl formula in a cubic simulation cell containing 62, 126 and 512 water molecules, corresponding to concentrations of 0.85, 0.43 and 0.11 M (mol litre^{-1}), respectively. The mean absolute error (MAE) between the results obtained with 62-water and 512-water cell for the 1D FES shown in Supplementary Figure 1(a), is about 0.032 kcal mol^{-1}; the error between results obtained with 126-water and 512-water cell is 0.028 kcal mol^{-1}. For the 2D FESs reported in Supplementary Figure 1(c-e), the MAE between 62-water and 512-water cell is about 0.27 kcal mol^{-1}, and that between 126-water and 512-water cell is 0.05 kcal mol^{-1}. Supplementary Figure 1(b) shows the Minimum free energy paths (MFEPs) obtained with

different simulation cells and they are nearly indistinguishable. These calculations show that the same FESs are obtained within a range of concentrations varying between 0.11 and 0.85 M.

Supplementary Note 2. Convergence of free energy surface at extreme conditions

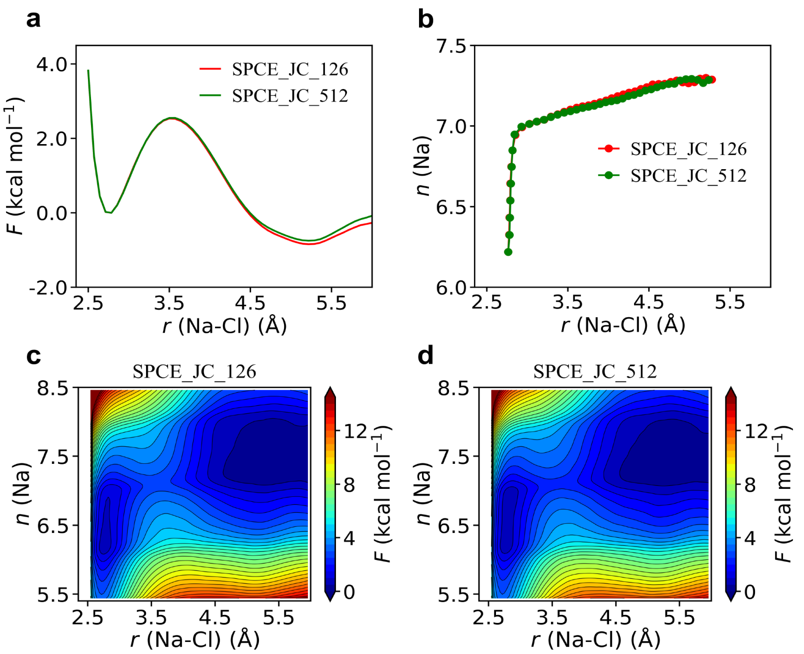

Supplementary Figure 2. Convergence of free energy surface with system size at extreme conditions. One (a, $F(r)$) and two (c-d, $F(r,n)$) dimensional free energy surfaces (FES) obtained using the SPCE_JC force-field at 1000 K and 11 GPa, using different simulation cell sizes. (b) Minimum free energy paths obtained for the 2D FESs shown in (c-d). r is the Na-Cl distance and n is the Na-water coordination number.

Using MD simulations with the SPCE_JC force-field, we tested the convergence of free energy surfaces (FES) with respect to salt concentration (cell size) at 1000 K and 11 GPa. Supplementary Figure 2 shows the FESs calculated for cells with one NaCl formula in a cubic simulation cell containing 126 and 512 water molecules, corresponding to a concentration of 0.68 M and 0.17 M respectively. For the 1D FESs in Supplementary Figure 2(a), the MAE between 126-water and 512-water cell is about 0.044 kcal mol^{-1}, and for the 2D FESs in Supplementary Figure 2(c-d), the MAE between 126-water and 512-water cell is about 0.18 kcal mol^{-1}. Supplementary Figure 2(b) shows that the MFEPs obtained with different cell sizes are nearly indistinguishable. These calculations show that the same FESs are obtained within a range of concentrations varying between 0.17 and 0.68 M. The 126-water cell is used for our FPMD simulations in the main text.

Supplementary Note 3. Convergence of free energy surface with simulation time

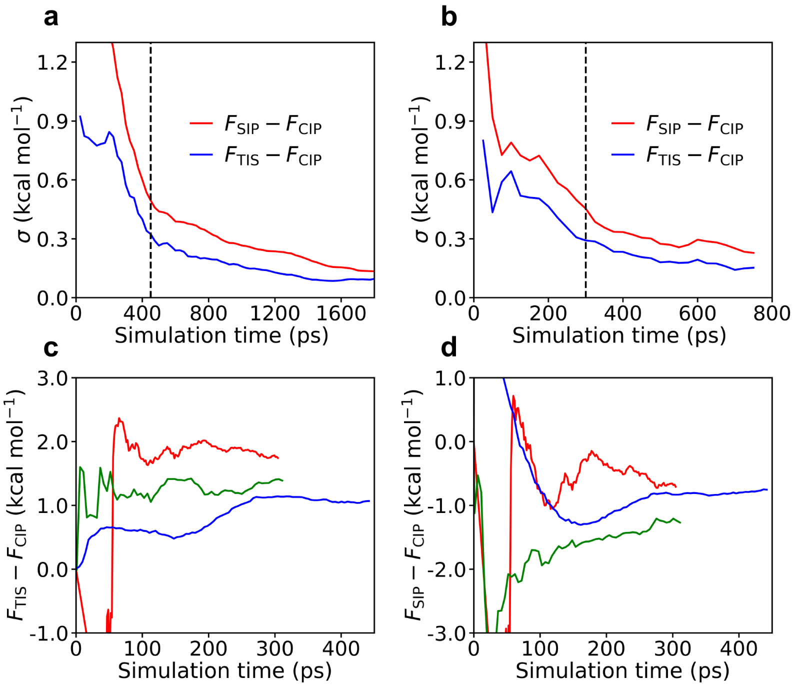

Supplementary Figure 3. Convergence of free energy surface with simulation time. The standard deviation (σ) of free energy difference ($F_{\mathrm{SIP}} - F_{\mathrm{CIP}}$ and $F_{\mathrm{TIS}} - F_{\mathrm{CIP}}$) obtained with 15 independent simulations using the SPCE_JC force-field at 300 K and 1 atm (a) and 1000 K and 11 GPa (b). The free energy difference $F_{\mathrm{TIS}} - F_{\mathrm{CIP}}$ (c) and $F_{\mathrm{SIP}} - F_{\mathrm{CIP}}$ (d) for our first principle calculations: PBE simulation at 1000 K and 11 GPa (red line), PEB simulation at 1000 K and 11 GPa with O-H bond fixed to 1 Å (green line) and PBE simulation at 400 K and 1 atm (blue line).

Using classical MD with the SPCE_JC force-field, we tested the convergence of the computed FESs with respect to simulation time at both ambient and extreme conditions. Supplementary Figure 3(a) and (b) show the results obtained from 15 independent simulations. We found that with 450 ps (300 ps) simulations (indicated by vertical dashed lines in the figure), the standard deviation of the relative free energy between CIP and SIP configurations ($F_{\mathrm{SIP}} - F_{\mathrm{CIP}}$) is $\sim$ 0.49 (0.45) kcal mol^{-1}, and that between TIS and CIP ($F_{\mathrm{TIS}} - F_{\mathrm{CIP}}$) is $\sim$ 0.32 (0.29) kcal mol^{-1} at ambient (high) $P - T$ conditions.

In Supplementary Figure 3(c) and (d), we plot the relative free energies $F_{\mathrm{TIS}} - F_{\mathrm{CIP}}$ and $F_{\mathrm{SIP}} - F_{\mathrm{CIP}}$ obtained from our first principle simulations.

Supplementary Note 4. Weighting scheme

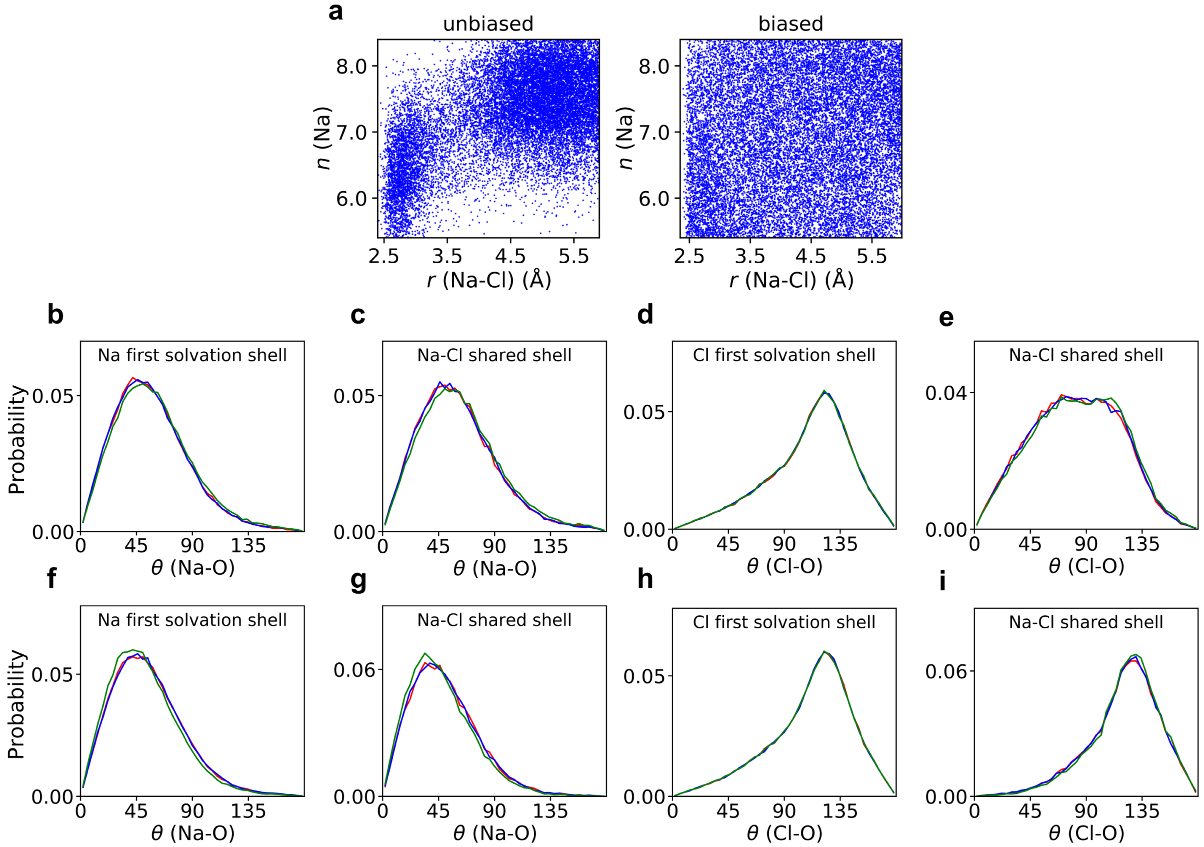

Supplementary Figure 4. Weighting scheme for biased MD simulations with the SPCE_JC force-field at 1000 K and 11 GPa. (a) Distribution of configurations during unbiased and biased MD simulations in Collective-Variable space: r is the Na-Cl distance and n is the Na-water coordination number. Probability distribution of angles (θ) between Na- and Cl-O bonds and the dipole of water molecules belonging to the ion first solvation shell (b,d,f,h), and the dipole of water molecules belonging to the shared Na-Cl shell (c,e,g,i) in the CIP (b-e) and SIP (f-i) configurations. We show angular distributions calculated from unbiased MD (red line), biased MD (green line) and the weighted results (blue line), using biased MD trajectories. The cutoff value used to define the first solvation shell is 3.2 (3.9) Å for Na^+ (Cl^-) ion.

As expected, biased and unbiased MD trajectories yield different probability distributions, as shown in Supplementary Figure 4(a). To extract physical quantities using biased trajectories, we used a weighting scheme. For configurations with Na-Cl distance r and Na-water

coordination number n, the following weights are assigned to the sampled configurations: $w(r,n) = C_0 \exp(-F(r,n)/k_\mathrm{B}T)$, where C_0 is a normalization constant. For example, to obtain $g(r)$, we need to first calculate $g(r,n)$ using only configurations corresponding to (r,n), then with $g(r)$ computed as:

$$g(r) = \int w(r,n) * g(r,n)\, dn \tag{1}$$

We first computed the angular distributions using configurations corresponding to different n and then we calculated the weighted distribution using the equation above.

We compared the angular distributions obtained directly from biased, unbiased MD trajectories and the weighted results (Supplementary Equation 1) for ion-pair in the CIP and SIP configurations (Supplementary Figure 4(b-i)). We found that the weighted distributions are in better agreement with those computed form unbiased MD simulations than the biased results. However, we noticed the angular distributions are not sensitive to the variation in Na-water coordination number, as even biased results are close to the unbiased ones.

Supplementary Note 5. Angular distribution of water dipole in NaCl solvation shell in constrained and unconstrained water simulations

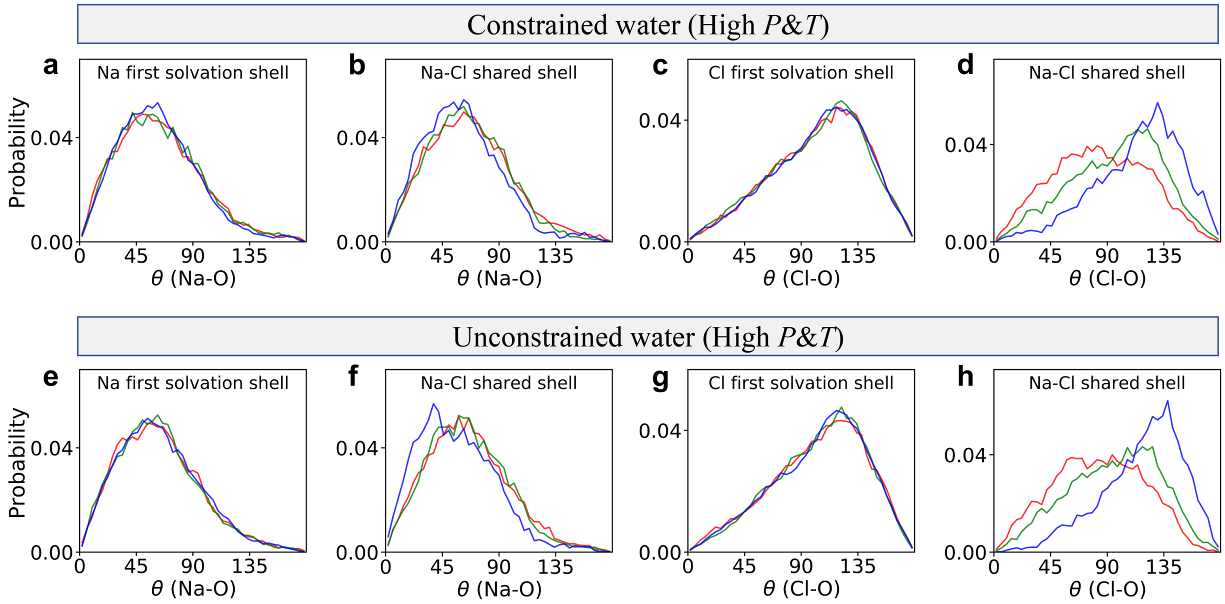

Supplementary Figure 5. Angular distribution functions. The probability distributions of angles (θ) between Na- and Cl-O bonds and the dipole of water molecules belonging to the ion first solvation shell (a,c,e,g), and the dipole of water molecules belonging to the shared Na-Cl shell (b,d,f,h), obtained with first principles molecular dynamics simulations. Panels (a-d) and (e-h) present the results obtained for CIP (red line), TIS (transition state, green line) and SIP (blue line) with O-H bonds of water being fixed and flexible at 1000K and 11 GPa, respectively. The cutoff value used to define the first solvation shell is 3.2 (3.9) Å for Na^+ (Cl^-) ion, chosen as the first minima of the ion-O radial distribution function.

Supplementary Note 6. Orientation of water in NaCl solvation shell at ambient and extreme conditions

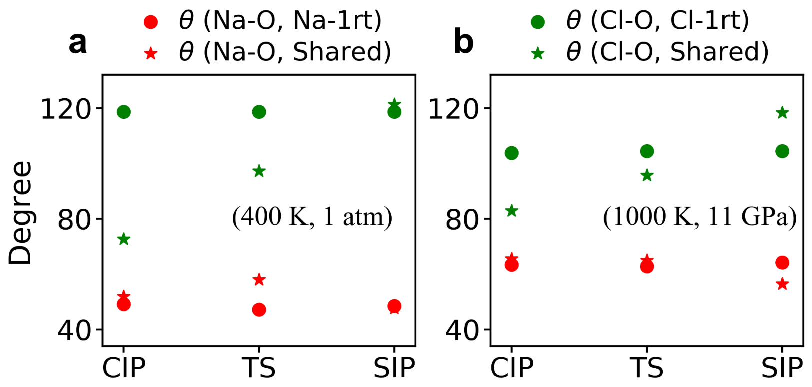

Supplementary Figure 6. Mean angles between water dipoles and ions. Calculated results using FPMD trajectories at ambient (a) and high $P-T$ (b) conditions. θ(Na-O, Na-1rt): angles between the Na-O bond and dipole of water in the first solvation sell of Na^+; θ(Cl-O, Cl-1rt): angles between the Cl-O bond and dipole of water in the first solvation sell of Cl^-; θ(Na-O, Shared): angles between the Na-O bond and dipole of water in the first solvation sell of both $Na^+$and Cl^-; θ(Cl-O, Shared): angles between the Cl-O bond and dipole of water in the first solvation sell of both $Na^+$and Cl^-. The cutoff value used to define the first solvation shell is 3.2 (3.9) Å for Na^+ (Cl^-) ion, chosen as the first minima of the ion-O radial distribution function.

The cell with one NaCl formula unit and 80 (126) water molecules is used for first principle calculations at 400 K and 1 atm (1000 K and 11 GPa), corresponding to concentration of ~0.68 M.

Supplementary Note 7. Angular distribution of water molecules in NaCl solvation shell at ambient and extreme conditions

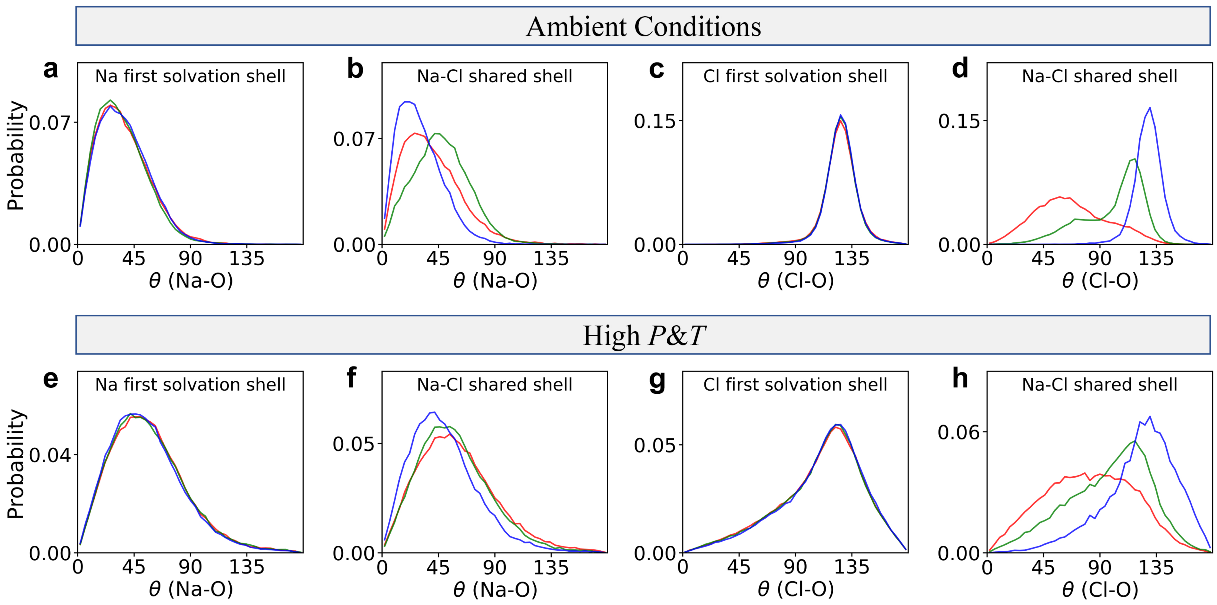

Supplementary Figure 7. Angular distribution functions obtained with SPCE_JC force-field. The probability distributions of angles (θ) between Na- and Cl-O bonds and the dipole of water molecules belonging to the ion first solvation shell (a,c,e,g), and the dipole of water molecules belonging to the shared Na-Cl shell (b,d,f,h). Panels (a-d) and (e-h) present the results obtained for CIP (red line), TIS (green line) and SIP (blue line) at 300 K and 1 atm and 1000K and 11 GPa, respectively. The cutoff value used to define the first solvation shell is 3.2 (3.9) Å for Na^+ (Cl^-) ion.

The cell with one NaCl formula unit and 126 water molecules is used for calculations at 300 K and 1 atm (1000 K and 11 GPa), corresponding to a concentration of $\sim$ 0.43 M ($\sim$ 0.68 M).

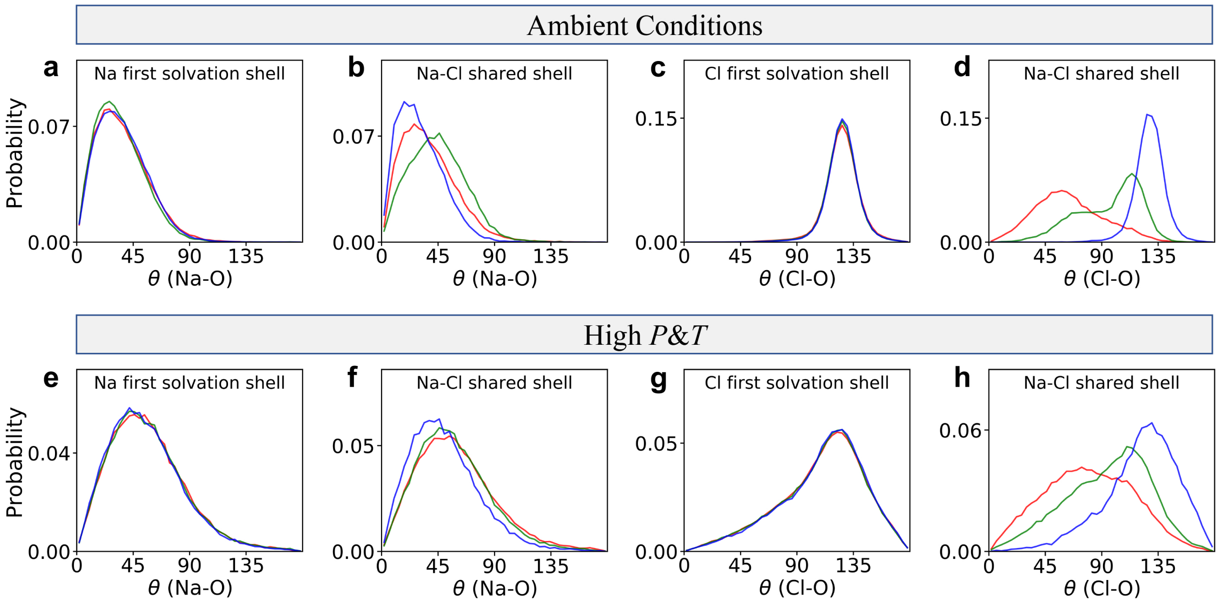

Supplementary Figure 8. Angular distribution functions obtained with SPCE_SD force-field. The probability distributions of angles (θ) between Na- and Cl-O bonds and the dipole of water molecules belonging to the ion first solvation shell (a,c,e,g), and the dipole of water molecules belonging to the shared Na-Cl shell (b,d,f,h). Panels (a-d) and (e-h) present the results obtained for CIP (red line), TIS (green line) and SIP (blue line) at 300 K and 1 atm and 1000K and 11 GPa, respectively. The cutoff value used to define the first solvation shell is 3.2 (3.9) Å for Na^+ (Cl^-) ion.

Supplementary Note 8. Ion-water coordination number

Supplementary Table 1. The coordination number of ions with water Oxygen at 300 K (400 K for PBE) and 1 atm and 1000 K and 11 GPa (in brackets).

	PBE		SPCE_JC		SPCE_SD		RPOL_SD	
	Na^+	Cl^-	Na^+	Cl^-	Na^+	Cl^-	Na^+	Cl^-
CIP	4.0 (6.4)	6.4 (12.6)	4.4 (6.6)	7.1 (12.9)	4.3 (6.5)	7.0 (13.1)	4.3(6.5)	6.9 (13.3)
SIP	5.2 (7.6)	6.9 (12.9)	5.8 (7.9)	7.3 (13.3)	5.8 (7.9)	7.3 (13.5)	5.8 (7.9)	7.5 (13.8)

The cell with one NaCl formula unit and 80 (126) water molecules is used for first principles calculations using PBE functional at 300 K and 1 atm (1000 K and 11 GPa), corresponding to a concentration of ∼0.68 M. While, one NaCl formula unit and 126-water system is employed for empirical MD simulations, corresponding to concentrations of ∼ 0.43 and 0.68 M at ambient and extreme conditions, respectively. The cutoff value used to define the first solvation shell is 3.2 (3.9) Å for Na^+ (Cl^-) ion. The results show that the coordination number of ions increase at high $P - T$; this increase is slightly overestimated by force-fields, compared to first principles simulations.

Supplementary Note 9. Two-dimensional free energy surface at ambient conditions

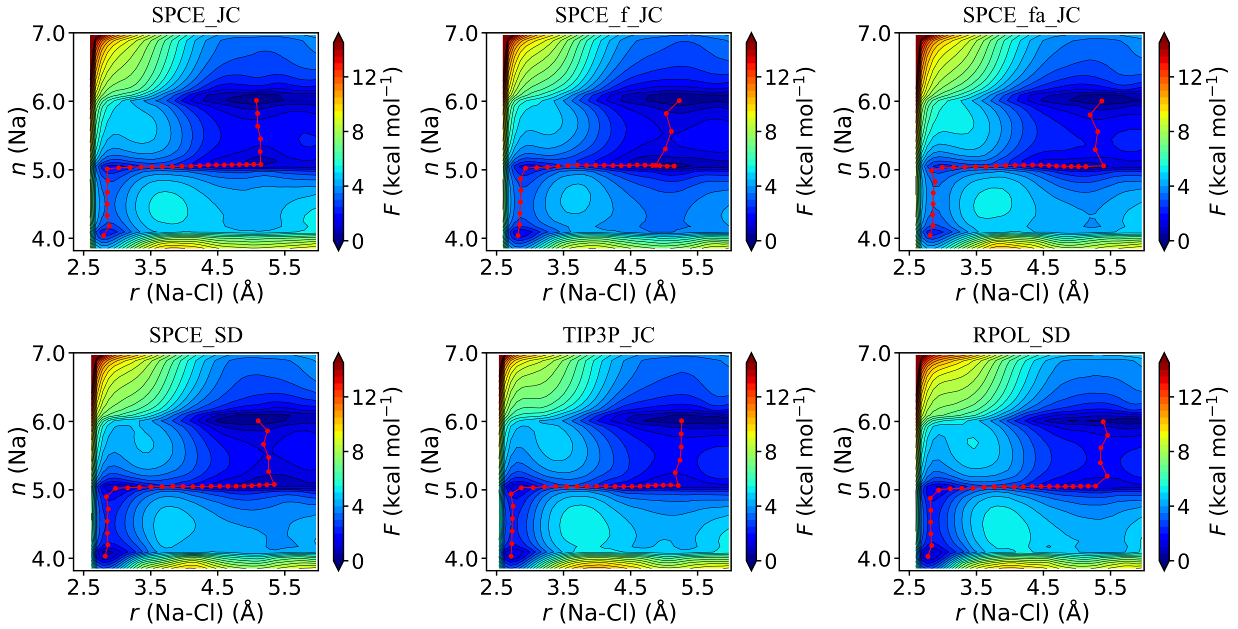

Supplementary Figure 9. Two-dimensional free energy surfaces $F(r, n)$ obtained with different force-fields and the corresponding MFEPs. r is the Na-Cl distance and n is the Na-water coordination number.

The cell with one NaCl formula unit and 126 water molecules ($\sim$ 0.43 M) is used for these calculations, and the free energy surfaces obtained with different force-fields display similar features.

Supplementary Note 10. Two-dimensional free energy surface at extreme conditions

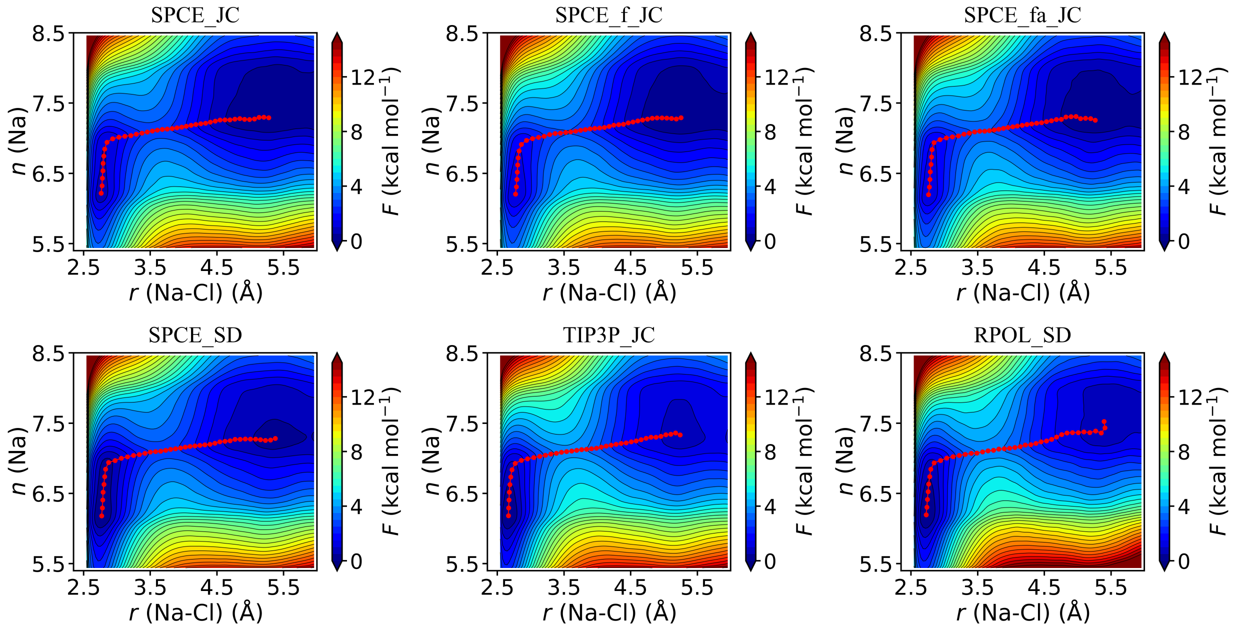

Supplementary Figure 10. Two-dimensional free energy surfaces $F(r, n)$ obtained with different force-fields and the corresponding MFEPs. r is the Na-Cl distance and n is the Na-water coordination number.

The cell with one NaCl formula unit and 126 water molecules (0.68 M) is used for these calculations, and the free energy surfaces obtained with different force-fields display similar features.

Supplementary Note 11. One-dimensional free energy surface at ambient and extreme conditions

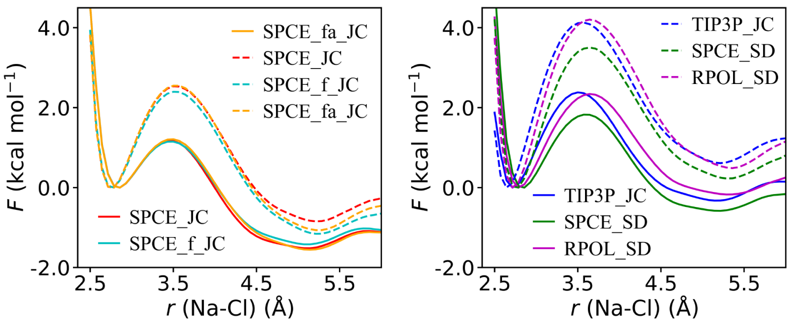

Supplementary Figure 11. Free energy surfaces $F(r)$ obtained with different force-fields at 300 K and 1 atm (solid line) and 1000 K and 11 GPa (dash line). r is the Na-Cl distance.

The cell with one NaCl formula unit and 126 water molecules is used for these calculations. The free energy surfaces obtained with different force-fields show an increased dissociation barrier and enhanced stability of CIP at 1000 K and 11 GPa.

Supplementary Note 12. Probability distribution at ambient and extreme conditions

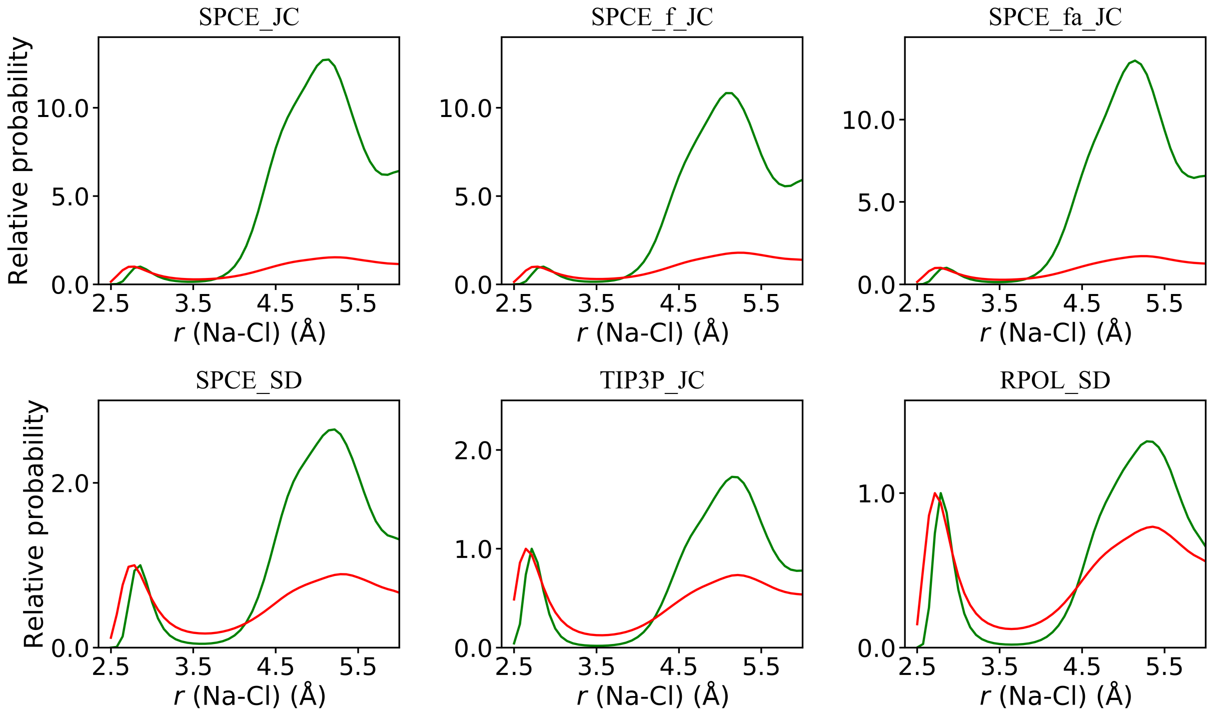

Supplementary Figure 12. The probability distribution of ion-pair obtained with force-fields. The results at 300 K and 1 atm (green line) and 1000 K and 11 GPa (red line) have been aligned at CIP: $\exp(-(F(r) - F_{\text{CIP}})/k_{\text{B}}T$. F is the free energy, T is the temperature, r is the Na-Cl distance and k_{B} is the Boltzmann constant.

The cell with one NaCl formula unit and 126 water molecules is used for these calculations. The probability distributions obtained with different force-fields show an increased stability of CIP at 1000 K and 11 GPa, consistent with the free energy surface changes reported in Supplementary Figure 11.
